# Genipin and insulin combined treatment improves implant osseointegration in type 2 diabetic rats

**DOI:** 10.1186/s13018-021-02210-1

**Published:** 2021-01-14

**Authors:** Jiajia Zhang, Ya-nan Wang, Tingting Jia, Haiyun Huang, Dongjiao Zhang, Xin Xu

**Affiliations:** Department of Implantology, School and Hospital of Stomatology, Cheeloo College of Medicine, Shandong University & Shandong Key Laboratory of Oral Tissue Regeneration & Shandong Engineering Laboratory for Dental Materials and Oral Tissue Regeneration, No. 44-1, Wenhua Road West, Jinan, 250012 Shandong Province China

**Keywords:** Type 2 diabetes mellitus (T2DM), Genipin, Insulin, Osseointegration, Oxidative stress, Adenosine 5′-monophosphate-activated protein kinase (AMPK)

## Abstract

**Background:**

Type 2 diabetes mellitus (T2DM) has a harmful effect on the stability and osseointegration of dental implants. T2DM induces mitochondrial damage by inhibiting AMPK signaling, resulting in oxidative stress and poor osteogenesis in the peri-implant bone area. Genipin is a major component of gardenia fruits with strong antioxidant, anti-inflammation, and antidiabetic actions, and it also can activate mitochondrial quality control via the AMPK pathway. The purpose of this study was to investigate the effects of genipin and insulin treatment on implant osseointegration in T2DM rats and explore the underlying mechanisms.

**Methods:**

Streptozotocin-induced diabetic rats received implant surgery in their femurs and were then assigned to five groups that were subjected to different treatments for three months: control group, T2DM group, insulin-treated T2DM group (10 IU/kg), genipin-treated T2DM group (50 mg/kg), and the genipin and insulin combination-treated T2DM group. Then, we regularly assessed the weight and glucose levels of the animals. Rats were euthanized at 3 months after the implantation procedure, and the femora were harvested for microscopic computerized tomography analysis, biomechanical tests, and different histomorphometric assessment.

**Results:**

The results indicated that the highest blood glucose and oxidative stress levels were measured for the T2DM group, resulting in the poorest osseointegration. The combination-treated T2DM group mitigated hyperglycemia and normalized, reactivated AMPK signaling, and alleviated oxidative stress as well as reversed the negative effect of osseointegration. There were beneficial changes observed in the T2DM-genipin and T2DM-insulin groups, but these were less in comparison to the combination treatment group.

**Conclusion:**

Our study suggests that treatment with genipin in combination with insulin could be an effective method for promoting implant osseointegration in T2DM rats, which may be related to AMPK signaling.

## Introduction

Type 2 diabetes mellitus (T2DM) is a prevalent metabolic disease characterized by abnormal regulation of glucose metabolism with diabetes-related complications, particularly cardiovascular disease, retinopathy, fragility fractures, osteoporosis, increased susceptibility to periodontal disease, and subsequent tooth loss [1–3]. With the constant development of surgical technology and implant design, dental restoration by the use of implants has become the first choice for edentulous or partially edentulous patients [4]. However, previous studies have demonstrated that T2DM adversely affects the bone regeneration and healing response around the implants [5, 6], leading to decreased success with oral implants for T2DM patients [7]. Others accept the fact that insulin is a prevailing trend in T2DM treatment, but it is restricted to improve all adverse effect on bone metabolism, and T2DM patients still exhibit poorer osteointegration of implants compared with healthy subjects [8, 9]. Therefore, it is imperative to explore new treatment modalities to enhance dental implant osseointegration in T2DM patients.

Genipin is an aglycone of the iridoid glycoside called geniposide that has diverse biological and pharmacological activities with strong antioxidant, anti-inflammation [10–12], and antidiabetic efficacies [13, 14]. Genipin has been used over the years in traditional oriental medicine to treat several inflammation-driven diseases and symptoms of type 2 diabetes mellitus [15, 16]. Moreover, genipin­crosslinked scaffolds, which have the capability to promote the proliferation, differentiation, and maturation of osteoblast­like cells, have been widely studied for bone tissue engineering [17].

AMPK is considered to be a key pivot of cellular metabolism that controls mitochondrial homeostasis [18, 19] and has significant effects on bone formation [20, 21]. Mitochondria are considered as the primary sites of reactive oxygen species (ROS) production [22, 23]. Recent studies have suggested that mitochondrial abnormalities in various tissues could lead to diabetes-related complications via increased production of ROS [24, 25]. Additionally, mitochondria serve as a pivotal modulator of differentiation, function, and survival of osteocytes [26, 27], osteoblasts [28, 29], and osteoclasts [30, 31] that influence bone formation. T2DM induces mitochondrial damage through inhibiting AMPK signaling, resulting in oxidative stress injury, dysfunction of osteoblasts, and poor osteogenesis in the peri-implant bone area [32, 33], while genipin can activate mitochondrial quality control via the AMPK pathway [34]. Furthermore, existing evidence showed that genipin could be a therapeutic candidate for the treatment of osteoporosis [35].

Therefore, this study aimed to investigate the hypothesis that long-term administration of genipin alone or combined with insulin might improve implant osteointegration in T2DM rats through inducing the reactivation of the AMPK pathway, with a final target being to conquer limitation of implantation in T2DM patients.

## Materials and methods

### Establishment of the T2DM rat model

All animal care and experimental protocols were strictly carried out according to international standards of animal welfare and in accordance with the Animal Ethics Committee of Shandong University (Jinan, China). Thirty male Sprague-Dawley rats (obtained from the Experimental Animal Center of Pengyue, Jinan, China) aged 10–11 weeks old with 200 ± 20 g body weight were chosen for this study. They were offered standard food and water ad libitum and were housed under a stabilized environment at a temperature of 25 °C, humidity of 55%, and with a 12:12-h light/dark cycle. After 7 days of acclimation to laboratory conditions, all rats were randomly divided into the control group (*n* = 6) or the type 2 diabetic groups (*n* = 24). Type 2 diabetic rats were induced by a 4-week high-fat and high-carbohydrate diet (53% carbohydrate, 31% total kcal of fat, and 16% protein; Beijing Ke’ao Xieli Feed Co. Ltd., China), followed by one intraperitoneal (IP) injection of streptozotocin (STZ, Sigma, USA) dissolved in 0.1 M citrate-buffered saline (pH 4.2) at a dose of 30 mg/kg [36]. The control rats received regular food and citrate buffer injection. Fasting blood glucose above 11.1 mmol/L steady for 1 week indicated successful establishment of the T2DM model.

### Implantation procedure

**Fig. 1:**
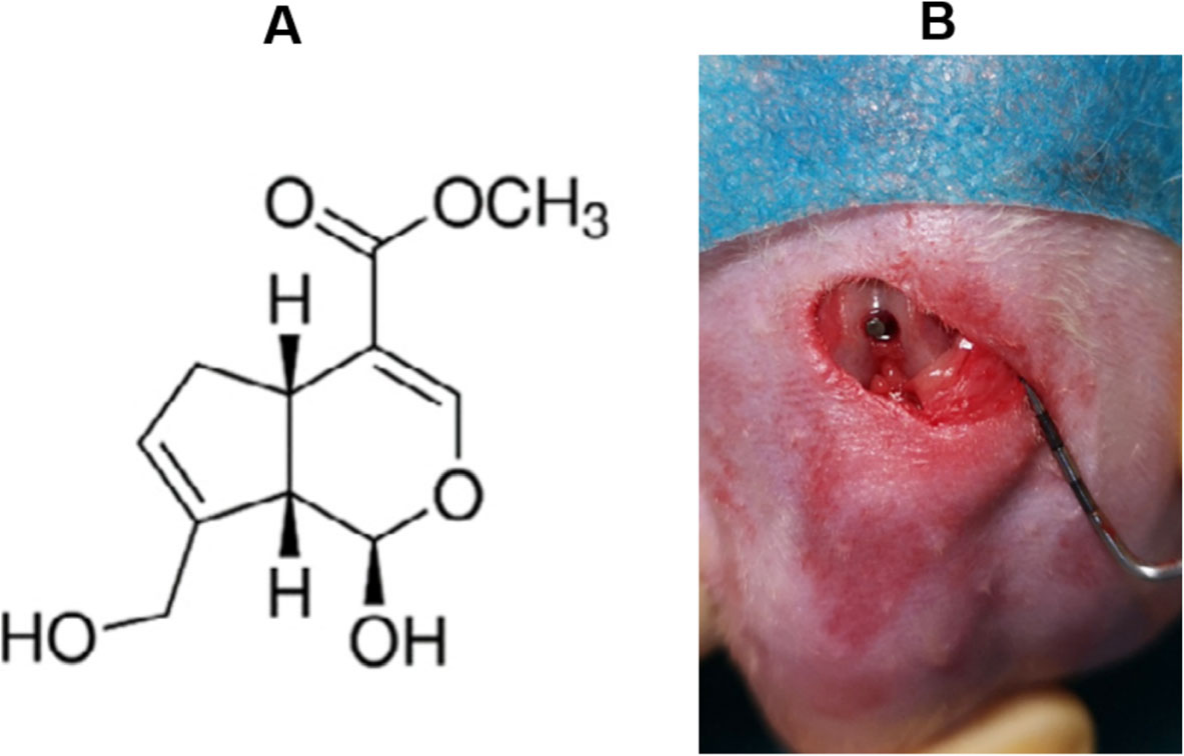
**a** The chemical structure of genipin. **b** The implant was introduced into the femoral medullary canal till the implant end was below the articular surface

The diabetic rats were randomly assigned into the following groups (with 6 rats per group): T2DM group, insulin-treated T2DM group (T2DM-insulin), genipin-treated T2DM group (T2DM-genipin), genipin and insulin combination-treated T2DM group (T2DM-genipin + insulin), and normal rats defined as the control group (*n* = 6 for every group). The chemical structure of genipin is displayed in Fig. 1a [37]. Animals were anesthetized by intraperitoneal injections of pentobarbital sodium (40 mg/kg body weight, Sigma, USA). Afterwards, every rat received two devised sand-blasted and acid-etched (SLA) titanium implants (1 mm in diameter and 10 mm in length) in the distal femurs as previously described [38]. By using a scalpel, a 1-cm-long incision was made along the medial side of the knee joint, and the extensor mechanism with the knee joint was laterally dislocated to expose the distal femoral metaphysis. With the knee in flexion, an implant bed was prepared from the intercondylar notch of the femur to access the medullary cavity with a rotary drill. Then, the implant was introduced into the femoral medullary canal until the implant end was below the articular surface (Fig. 1b). The extensor mechanism was relocated, and the surgical incisions were closed in layers. All the rats received intramuscular injection of antibiotic immediately after surgery and for three postoperative days.

### Treatment regimens

Three days after implantation, neutral protamine Hagedorn insulin (10 IU/kg, Novolin, Denmark) was administered by subcutaneous injection every day for the T2DM-insulin group during the entire experiment [39]. The T2DM-genipin rats were treated for 12 weeks with genipin (Nanjing Dilger Medical Technology Co. Ltd., China, 98% purity), which was dissolved in dimethyl sulfoxide (DMSO) and diluted in saline solution to a final concentration of 1% DMSO, at a dose of 50 mg/kg/day by oral gavage. The dosage and timing of genipin administration were determined based on previous studies [40, 41]. Rats in the T2DM-genipin + insulin group were treated with genipin and insulin daily. In the control group and T2DM group, DMSO and saline solution were administered using the same volume and route as those of the genipin treatment group. Body weight and fasting blood glucose determined from caudal vein blood in different groups were regularly monitored. Three months after surgery, all rats were euthanized, and the double-sided femora with implants were further evaluated.

### Microscopic computerized tomography (micro-CT) reconstruction

After soft tissue was removed, the non-demineralized specimens were scanned on a micro-CT system (60 kV/5 W, Quantum GX2, PerkinElmer, Japan), and three-dimensional (3-D) images were reconstructed from the microtomographic slices. The region of interest (ROI) was defined as an annular domain of the 200-μm region around the implant. The parameters of bone volume per total volume (BV/TV), the mean trabecular number (Tb.N), and percentage of osseointegration (OI%), which was represented as the percentage of bone voxels to total voxels in direct contact with the implant, were analyzed within the ROI zone in order to assess osteogenesis associated with the implants.

### Pull-out test

The pull-out test [42] can evaluate the biomechanical properties of the implant-bone interfaces. After specimens were collected, the test was performed using a universal mechanical testing machine (Shimadzu, Japan) at a loading rate of 1 mm/min until the implant-bone interface ruptured. The maximum retention force gained by the implant in the bone, which is referred to as the maximal pulling force, was recorded.

### Histology and histomorphometric analysis

#### Hard tissue slicing

Specimens including implants were dehydrated through a series of graded ethanol solutions, embedded in methylmethacrylate without decalcification, and sawn using a rotary diamond saw (SP1600, Leica, Germany). Thereby, we obtained undecalcified 50-μm-thick sections, which were stained in methylene blue-acid fuchsin. Bone-to-implant contact (BIC, the length percentage of the direct interface of bone and implant to the total implant’s surface in the cancellous bone) was calculated using the Image-Pro Plus 6.0 software [43].

#### Preparation and staining of decalcified sections

We obtained the specimens whose soft tissue was removed after the rats were euthanized. After fixation in 4% polyformaldehyde for 48 h and washing under flowing water, the femora were decalcified in 10% EDTA solution for 6–8 weeks until a needle was able to pierce the femur without resistance. Then, without damaging the interface tissue, we carefully removed the implants along the direction of the major axis, after which the femurs were dehydrated in gradient ethanol and embedded in paraffin. A slicer was used to produce 5-μm sections around the implants, in order to evaluate the formation and maturity of new bone by separately staining with hematoxylin-eosin (HE) and modified Masson.

#### Immunohistochemistry (IHC) examinations

For prepared decalcified sections, IHC staining was processed with primary antibodies against phospho-AMPK (CST, 2535 T, 1:100 dilution) and 8-OHdG (8-hydroxy-20-deoxyguanosine, a marker of DNA damage in oxidative stress, Santa Cruz Biotechnology, sc-393871, 1:200 dilution) as previously reported [32, 44]. For quantitative valuation, three slices from each group were observed using an optical microscope and analyzed by Image-Pro Plus 6.0. The mean integrated optical density (IOD) for pAMPK and 8-OHdG were also calculated around the implants.

### Statistical analysis

The statistical analyses are presented as the mean ± standard deviation (SD) of at least three independent measurements. One-way ANOVA was used to analyze group-to-group statistical differences. The significance level was set at *p* < 0.05. All calculations were performed using the SPSS software (SPSS 23.0).

## Results

### Measurement of body weight and fasting glucose levels

Changes in weight were recorded in the experiment (Table 1). The weights of all animals increased throughout the test except for the rats in the T2DM group. Generally, the body weights of the rats in the T2DM-genipin + insulin group increased in diabetes to the highest value compared with the T2DM-insulin and T2DM-genipin groups.

**Table 1:**
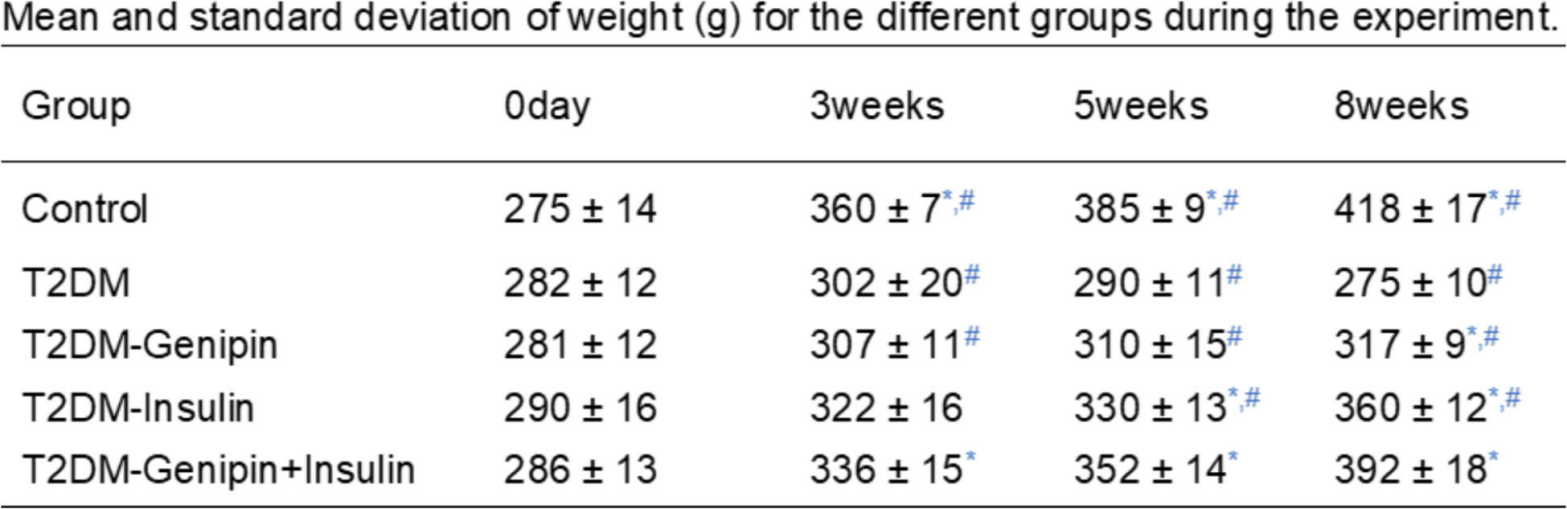
Mean and standard deviation of weight (g) for the different groups during the experiment

Zero day represented the day before STZ injection, 1 week represented the implantation time, 2–12 weeks represented the corresponding time after surgery. Data are expressed as mean ± SD, *n* = 6/group

*p < 0.05, for T2DM vs. others

^#^*p* < 0.05, for T2DM-genipin + insulin vs. others

**Fig. 2:**
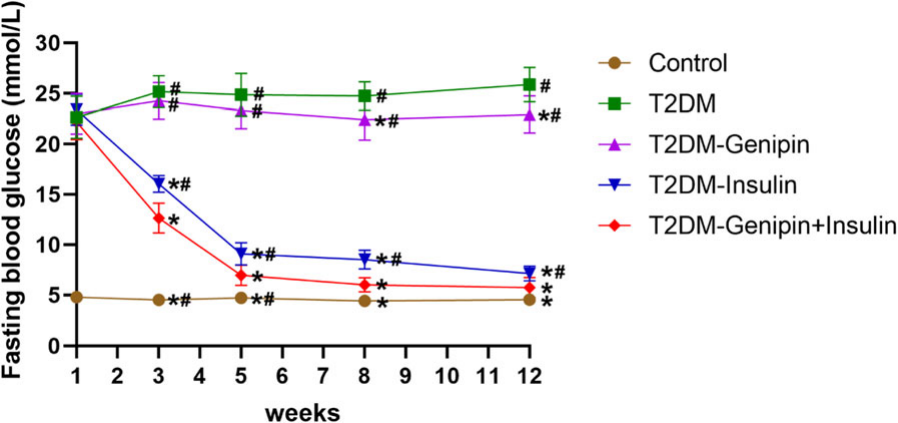
Fasting blood glucose (mmol/L) levels in experimental rats (*n* = 6/group). One week represented the implantation time, 2–12 weeks represented the corresponding time after surgery. Data are expressed as mean ± SD. *p < 0.05, for T2DM vs. others, #*p* < 0.05, for T2DM-genipin + insulin vs. others

Blood glucose levels of all animals are displayed in Fig. 2 over the period and were all within the normal range before STZ injection. But the glucose levels of the T2DM rats conspicuously increased compared to normal rats after STZ administration. Through treatment regimens as mentioned above, we could see a distinct hypoglycemic effect, especially in the combination-treated group. High glucose levels in the combination-treated group and T2DM-insulin group were found to be normal, but there were no discernible decreases in glucose levels in the T2DM-genipin group.

### Reconstruction and assessment of micro-CT

**Fig. 3:**
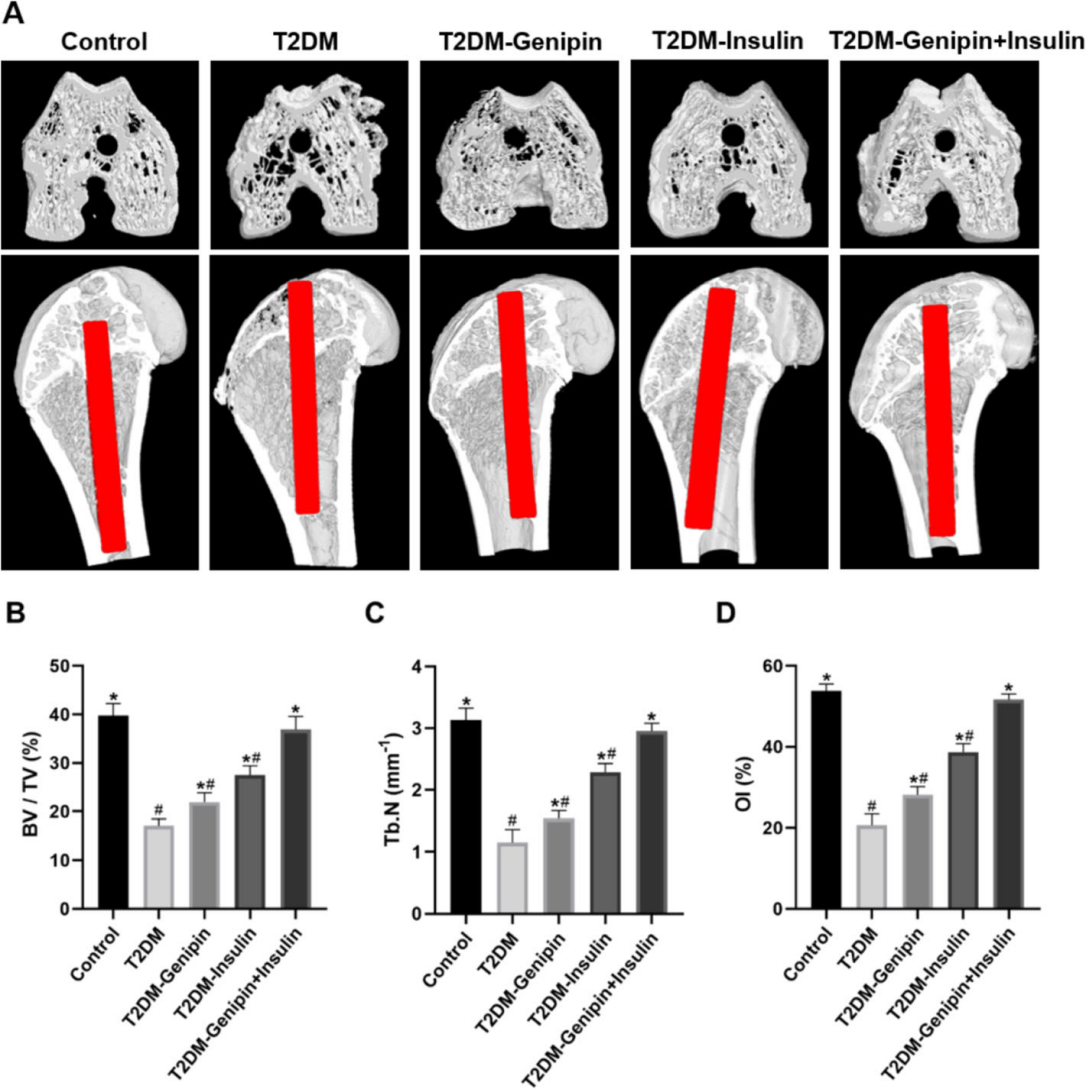
Results of micro-CT at the bone-implant interface (*n* = 4 specimens/group). **a** Micro-CT images of femurs with implants showed the bone (white) attached to the implant (red) after 3-month surgery: the upper row displayed 3D images of the coronary section through the longitudinal axis of implants, and the lower row demonstrated 3D images of the transverse section of femur implants. And the statistical results of BV/TV (**b**), Tb.N (**c**), and %OI (**d**) around the implants according to the micro-CT. Data are expressed as mean ± SD. *p < 0.05, for T2DM vs. others, #*p* < 0.05, for T2DM-genipin + insulin vs. others

Reconstructed 3D micro-CT images (Fig. 3a) showed that the T2DM group had few trabecular microstructure improvements with little osteogenesis. In diabetic rats, the combination-treated group exhibited the most optimal effects that resulted in improved trabecular microstructure and osteogenesis around the implant-bone interface. Quantitative analysis (Fig. 3b–d) provided more details on BV/TV, Tb.N, and OI%. Compared with the T2DM group, the combination-treated group exhibited increased BV/TV by 1.156-fold, Tb.N by 1.558-fold, and OI% by 1.500-fold (*p* < 0.05). However, we observed a negligible discrepancy between the combination-treated group and control group (*p* < 0.05). Additionally, there were increased values for the T2DM-genipin group and T2DM-insulin group, but they were inferior to those of the combined-treated group.

### Assessment of the pull-out test

**Fig. 4:**
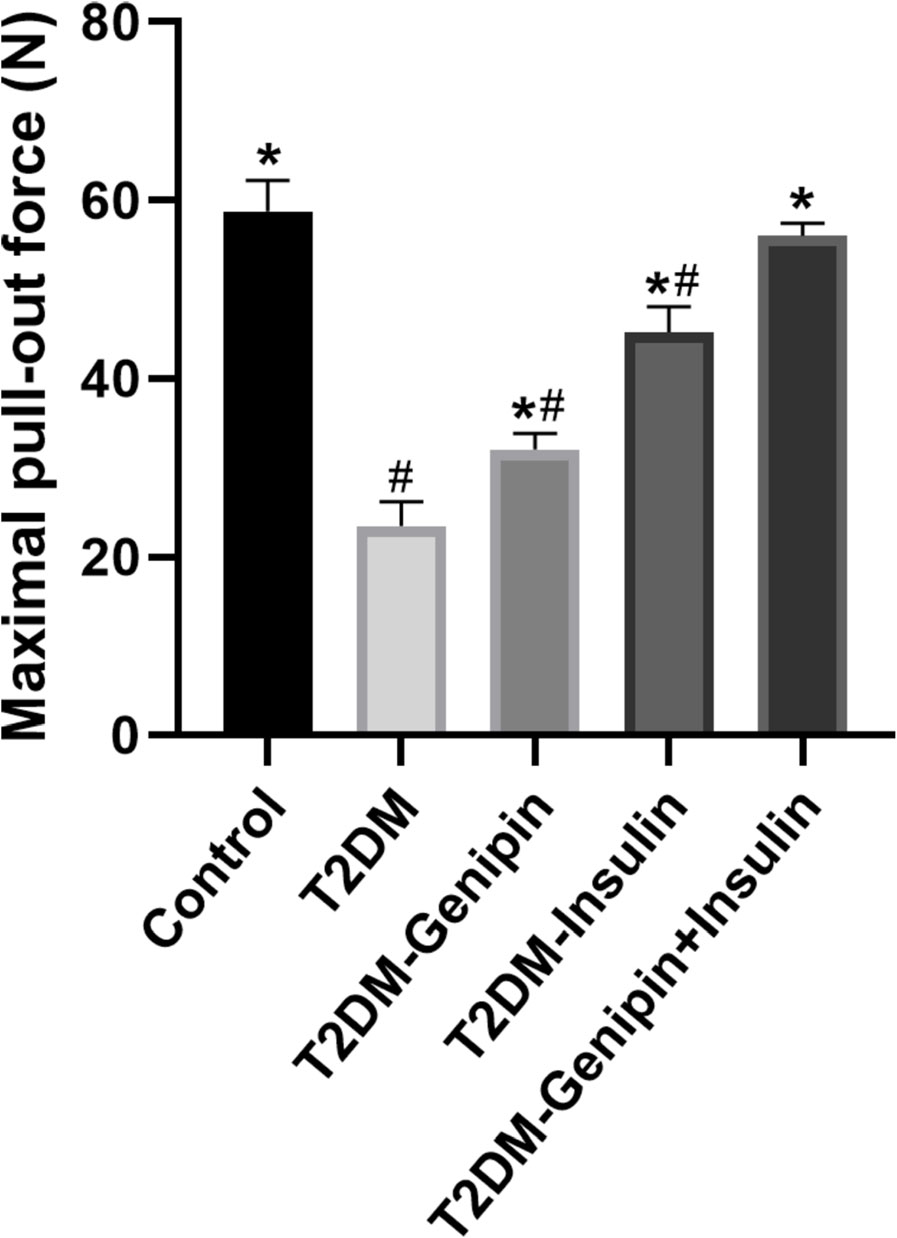
Histogram of the pull-out test at 3 months after implantation (*n* = 4 specimens/group). Data are expressed as mean ± SD. *p < 0.05, for T2DM vs. others, #*p* < 0.05, for T2DM-genipin + insulin vs. others

The outcome from the pull-out test is displayed in Fig. 4, and the results were similar to those obtained with micro-CT assessment. There were higher values for biomechanical tests in the combination-treated group and control group (*p* < 0.05). Compared with the T2DM group, the combination-treated group exhibited values that were significantly increased by 1.383-fold (*p* < 0.05). For the mono-treatment (of genipin or insulin), a positive function was also expressed, but to a lesser extent.

### Evaluation of histological and histomorphometric experiments

#### Hard tissue slicing

**Fig. 5:**
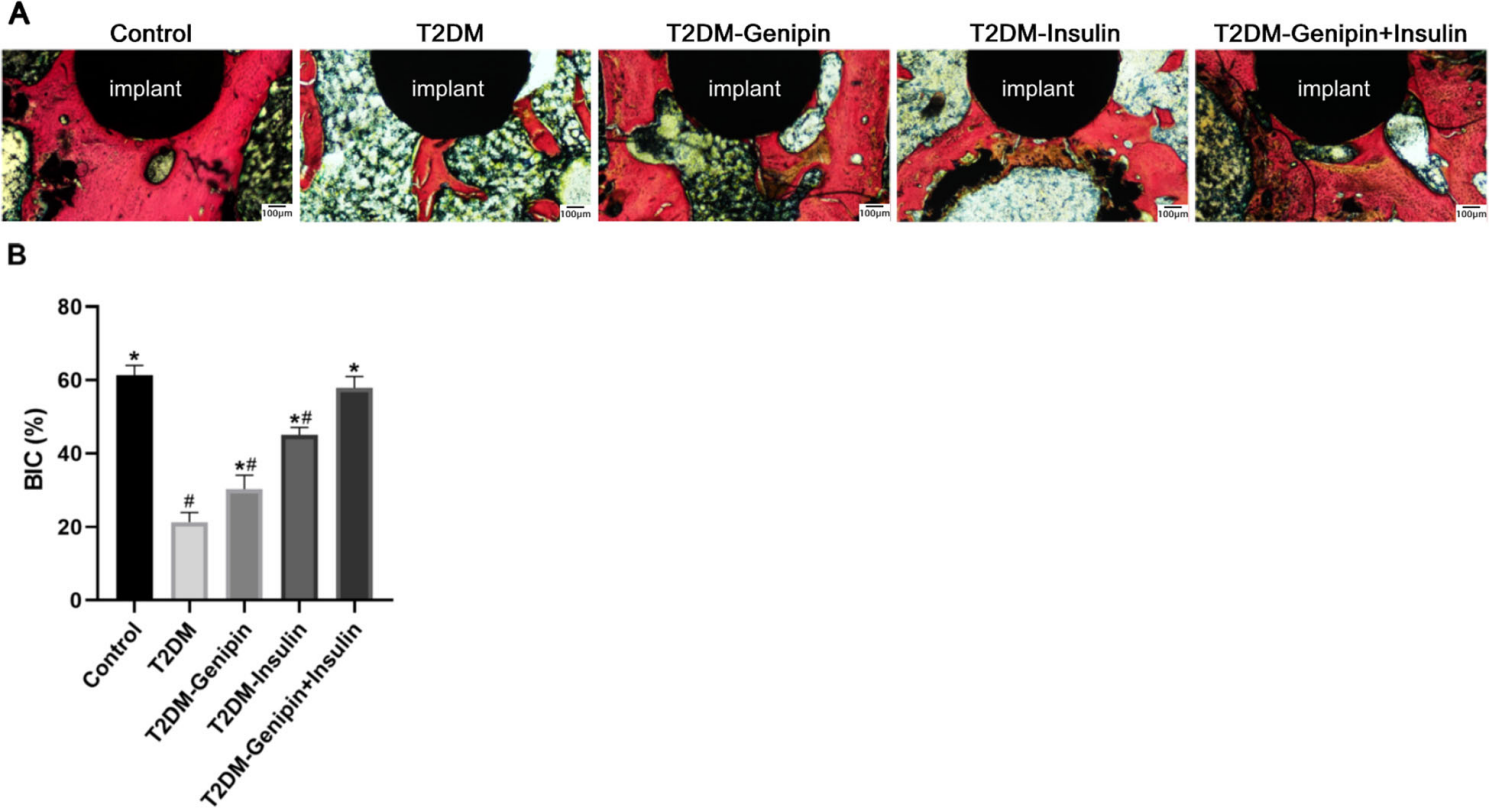
Results of hard tissue slicing at the bone-implant interface (*n* = 4/group). **a** Histological images of undecalcified sections by methylene blue-acid fuchsin staining. The implant is in the black area. **b** Quantitative analysis of the bone-implant contact ratio (BIC, %). Data are expressed as mean ± SD. *p < 0.05, for T2DM vs. others, #*p* < 0.05, for T2DM-genipin + insulin vs. others

The results of undecalcified section staining (Fig. 5a) were similar to those of micro-CT and also indicated that the combination-treated group increased their peri-implant bone mass in T2DM rats to the greatest degree. Quantitative analyses were displayed as BIC (Fig. 5b). Mono-genipin treatment moderately improved the histomorphometric parameters of implants, with the BIC increased by 0.415-fold in comparison to the T2DM group, but not more than the mono-insulin treatment. However, the T2DM group developed a BIC percentage of 21.4%, which was clearly less than that of the control group, which obtained 61.4% BIC (*p* < 0.05).

#### Hematoxylin-eosin and modified Masson staining

**Fig. 6:**
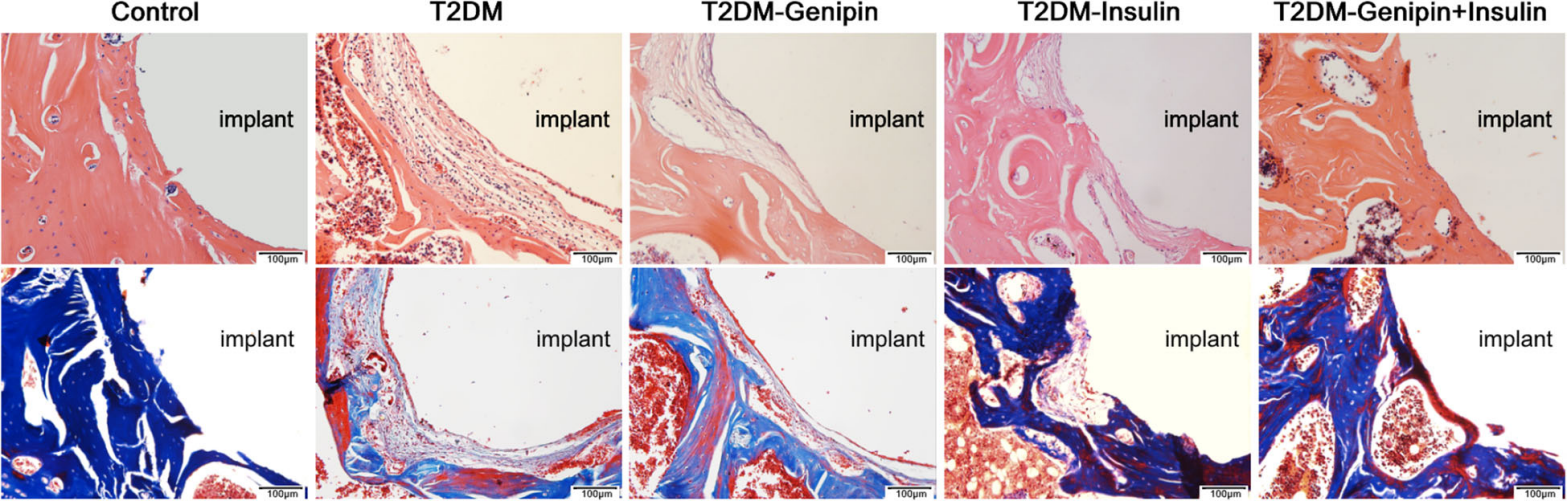
Bone microarchitectures were observed by representative HE staining (upper) and modified Masson staining (lower)

Hematoxylin-eosin staining (Fig. 6) showed that in the T2DM group, the area around the implants was nearly all filled with fibrous tissue. In contrast, different amounts of bone tissue were noted in the control and the three treated groups. The outcome of Masson staining (Fig. 6) indicated a red-blue color around implants that signified the presence of new bone, and the combination-treated group and controls further increased their rate of mature bone generation. We also observed that there was a stronger osteogenic effect in the T2DM-insulin group than that of the T2DM-genipin group, but not as strong as the combination-treated group and control group.

#### Immunohistochemistry staining

**Fig. 7:**
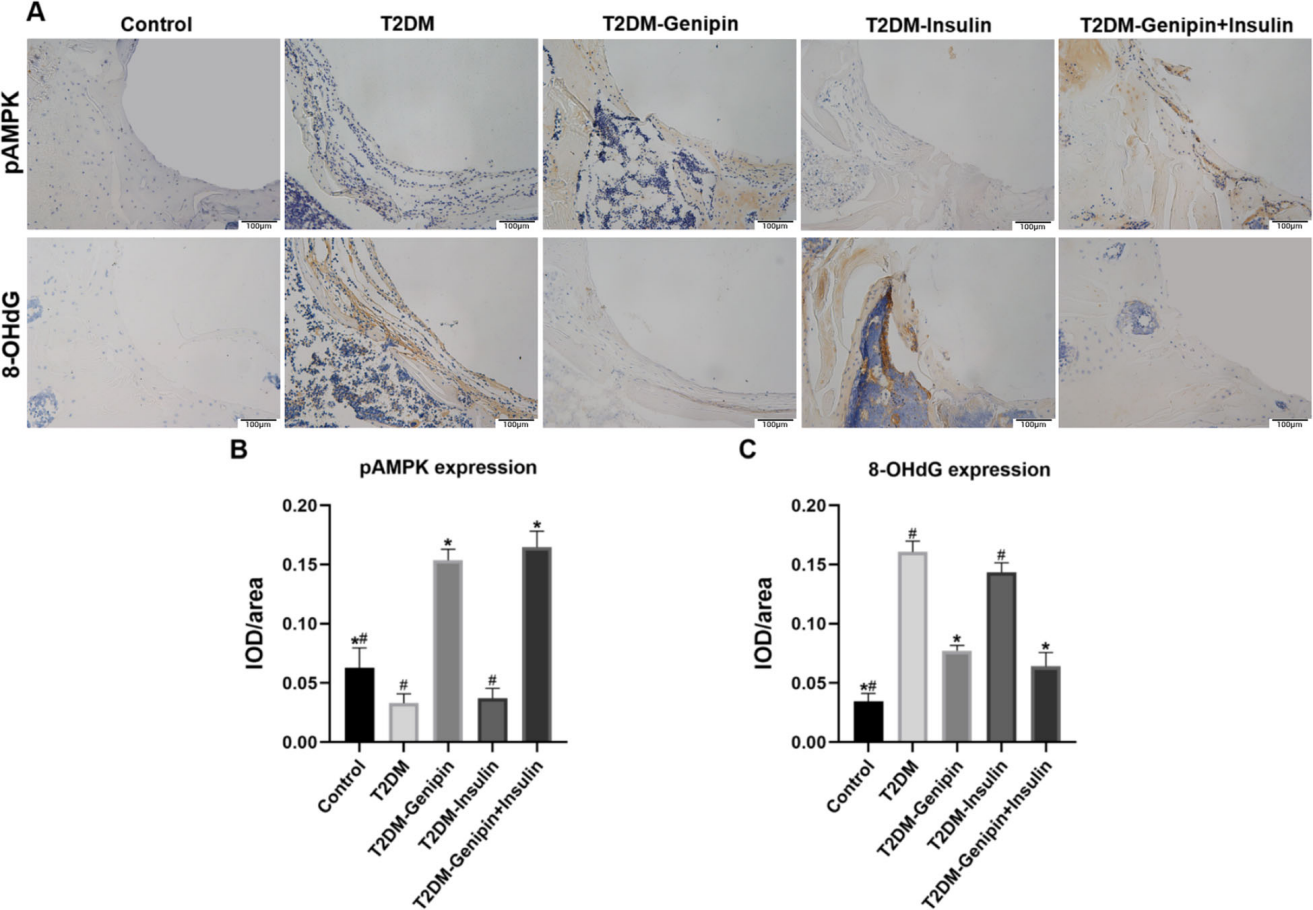
AMPK activation and oxidative stress levels in the peri-implant bone area (*n* = 3/group). **a** Representative immunohistochemical images of pAMPK and 8-OHdG (a marker of DNA damage caused by oxidative stress) around the implants. Quantitative analysis of mean integral optical density (IOD) for pAMPK **b** and 8-OHdG **c** in the ROI. Data are expressed as mean ± SD. *p < 0.05, for T2DM vs. others, #*p* < 0.05, for T2DM-genipin + insulin vs. others

We evaluated the level of AMPK activation and oxidative stress injury in the tissue around the implants by immunohistochemical staining of pAMPK and 8-OHdG (Fig. 7). Similar to previous results, the T2DM group exhibited lower pAMPK and higher 8-OHdG expression levels compared with the control group. However, the genipin treatment distinctly increased the AMPK activity and significantly decreased the concentration of 8-OHdG in T2DM rats. The IOD value further showed that genipin treatment could play its beneficial role by reactivating AMPK signaling and the antioxidant response.

## Discussion

It is well-known that diabetes mellitus (DM) remains a primary contraindication for the clinical application of titanium implants, which have poorer osteointegration and a higher failure rate in diabetics. However, based on the most recent International Diabetes Federation (IDF) report, the global DM prevalence is estimated to be 463 million individuals in 2019, and is predicted to increase to 700 million by the year 2045 [45]. Moreover, T2DM accounts for about 90% of DM [45].

There is increasing evidence that T2DM may impede bone regeneration of dental implants. The T2DM model in rats, induced by a high-fat diet and low-dose STZ, approximately embodies the metabolic characteristics of human T2DM, and results in enduring and steady hyperglycemia [46, 47]. We successfully built a T2DM model with lighter body weight and higher blood glucose levels due to the pancreatic β-cell dysfunction induced by STZ [48]. Our research also indicated impaired implant stability and reduced microstructure of the trabecula, as well as decreased osseointegration around the implants in the T2DM group, which is also in agreement with previous studies in this related field [32, 49].

Thus far, insulin administration remains the cornerstone of DM management [50], and earlier studies have shown that insulin therapy could dominate blood sugar and ameliorate bone regeneration of implants for diabetics [1, 51, 52]. However, the concrete effects are still debatable. Moreover, existing evidence indicated that insulin-treated DM rats had poorer bone regeneration and formation than normal controls [53, 54], which is in accordance with our results. In our study, hyperglycemia was reduced to a normal level, and body weights were elevated by insulin therapy. In addition, the pull-out test indicated that insulin treatment slightly increased implant stability in T2DM rats. The results from micro-CT and histomorphometric evaluation showed that the trabecular microstructure and bone regeneration of the insulin-treated T2DM group were improved to a certain extent, but there was no comparability to controls. Consistent with previous findings [9, 55], our study demonstrated that insulin alone was inadequate to reverse all the adverse effects of T2DM on implant osseointegration. Therefore, there is considerable demand in searching alternative or complementary methods that can be used to promote implant osseointegration in T2DM.

Genipin can ameliorate insulin sensitivity [14, 16], acutely reverse high-glucose-induced β-cell dysfunction, and stimulate insulin secretion [13]. Additionally, genipin can exert potent anti-inflammatory activity [10, 12] and powerful cytoprotective effects against ROS-induced cytotoxicity [56]. Existing evidence showed that genipin could be a therapeutic candidate for the treatment of osteoporosis [35]. Our study found that it had little effect on altering weight and fasting blood glucose in mono-genipin-treated rats. However, immunohistochemical staining showed that it increased AMPK reactivation and alleviated oxidative stress around the implants. Furthermore, the evaluation indicators of bone regeneration slightly improved in T2DM rats treated by only genipin.

Hence, our research aimed to probe the effect of genipin in combination with insulin treatment on implant osseointegration in T2DM rats and to discover whether the effect is accessional. As anticipated, the combined treatment resulted in recovery of blood sugar and weight to the normal range, and reversed the damaged bone regeneration around implants in T2DM rats. Moreover, micro-CT and histological evaluations contributed to interpreting the results of the pull-out test at the structural level, which confirmed our hypothesis. For quantitative assessment, micro-CT and histological analyses showed that the parameters of BIC%, BV/TV, Tb.N, and OI% were improved to the greatest extent in T2DM by the combined therapy. Taken together, it established that combined therapy ameliorated implant osteointegration of T2DM rats with increasing mass and density of bone. In addition, HE and Masson staining assessed the maturity and formation rate of new bone during the osteointegration process and showed consistent results.

Next, we further explored the underlying mechanism of genipin that resulted in an additive impact on promoting bone regeneration in T2DM rats. Recently, studies have shown that AMPK is a key regulator of mitochondrial quality through mechanisms such as increasing mitochondrial biogenesis [57] and eliminating impaired mitochondria by autophagy [58]. Additionally, AMPK is an important molecular target for metabolic diseases such as diabetes to enhance the recovery of osteoblast function and osseointegration around implants [32]. However, AMPK can be negatively regulated by high glucose levels [59], which initiate the complications of diabetes [60]. As previously described, T2DM induced mitochondrial damage by inhibiting AMPK signaling, resulting in oxidative stress injury and poor osteogenesis around implants [32, 33], while genipin could activate mitochondrial quality control and play a protective role by reactivating the AMPK pathway [34]. Our study also confirmed that the T2DM group had lower pAMPK expression levels and higher oxidative stress injuries compared to the control group, but the T2DM-genipin and combination-treated groups significantly increased AMPK activity and reduced oxidative stress injury that was beneficial for implant osseointegration. In addition to reactivating the AMPK pathway, the function of genipin may also be related to the recovery of the antioxidant response. The specific mechanisms require further exploration.

Taken together, our results confirmed that the combination treatment of genipin and insulin had positive influences on new bone formation and implant stability for T2DM rats 12 weeks after implantation. However, we still require longer study times to assess the enduring efficacy of genipin and insulin combined therapy.

## Conclusion

We proposed the pharmacological action of genipin on improving bone remodeling in T2DM rats. In conclusion, our findings collectively established that combined treatment with genipin and insulin had a synergistic or additive influence on reversing the poor osseointegration of implants in T2DM rats, and the potential mechanisms may be associated with AMPK signaling reactivation by genipin. However, it is uncertain whether the results from animal studies can be applied to humans, and therefore, further research is essential.

## Data Availability

The datasets used and/or analyzed during the current study are available from the corresponding author on reasonable request.
